# Skeletal Muscle MicroRNAs as Key Players in the Pathogenesis of Amyotrophic Lateral Sclerosis

**DOI:** 10.3390/ijms19051534

**Published:** 2018-05-22

**Authors:** Lorena Di Pietro, Wanda Lattanzi, Camilla Bernardini

**Affiliations:** Istituto di Anatomia Umana e Biologia Cellulare, Università Cattolica del Sacro Cuore, 00168 Rome, Italy; lorena.dipietro@unicatt.it (L.D.P.); wanda.lattanzi@unicatt.it (W.L.)

**Keywords:** amyotrophic lateral sclerosis, microRNA, skeletal muscle, gene expression signature, molecular biomarkers, molecularly targeted therapies

## Abstract

Amyotrophic lateral sclerosis (ALS) is a fatal neurodegenerative disorder, for which, to date, no effective treatment to ameliorate the clinical manifestations is available. The long-standing view of ALS as affecting only motor neurons has been challenged by the finding that the skeletal muscle plays an active role in the disease pathogenesis and can be a valuable target for therapeutic strategies. In recent years, non-coding RNAs, including microRNAs, have emerged as important molecules that play key roles in several cellular mechanisms involved in the pathogenic mechanisms underlying various human conditions. In this review, we summarize how the expression of some microRNAs is dysregulated in the skeletal muscle of ALS mouse models and patients. Shedding light on the mechanisms underlying microRNAs dysregulation in the skeletal muscle could clarify some of the processes involved in the pathogenesis of ALS and especially identify new promising therapeutic targets in patients.

## 1. Introduction

Amyotrophic lateral sclerosis (ALS) is a fatal neurodegenerative disorder, characterized by motor neuron degeneration, followed by muscle weakness, paralysis, and death. ALS is caused by a combination of genetic, epigenetic, and environmental risk factors and patients undergo a very variable disease progression, with death usually occurring because of respiratory failure in 3–5 years. Over 90% cases of ALS are sporadic, while the remaining 5–10% show a familial inheritance. More than 20 genes have been identified whose mutations are involved in the development of the disease. Mutations in *C9orf72*, *SOD1*, *TARDBP*, *UBQLN2*, and *FUS* genes are the most frequent. To date, no effective treatment to remarkably ameliorate the clinical manifestations is available. For a long time riluzole has remained the only treatment, offering modest survival benefit for ALS patients. Most recently, a second drug, edaravone, has been approved by the US Food and Drug Administration. Edaravone leads to a reduction of ALSFRS-R (ALS Functional Rating Scale Revised) score, while, to date, there are no data indicating any longer-term effect on patients’ safety and survival [1].

The long-standing view of ALS as affecting only motor neurons has been recently challenged by the finding that ALS is a multi-systemic disease, in which other cell types, beyond motor neurons, such as microglia, astrocytes, muscle, and T-cells, are involved in the pathogenesis of the disease. The pathological modifications in motor axons and nerve terminals precede motor neuron degeneration and the onset of clinical symptoms [2,3,4,5,6]; this indication has led to ALS being suggested as a distal axonopathy, whereby skeletal muscle contributes to a retrograde signaling cascade that degrades motor neurons [4,5,7,8].

Before the clinical onset and during the disease progression, the skeletal muscle of ALS patients undergoes futile cycles of reinnervation and denervation, along with motor neuron degeneration [9]. When the motor neurons die, the surviving neurons, in order to compensate for the missing synapses, reinnervate the muscle fibers, hence the skeletal muscle is reorganized by clustering fibers of the same metabolic type, giving rise to the phenomenon of “fiber type grouping”. Ultimately, these motor units lose their innervation and the atrophy process starts.

Muscle fibers are classified into two main metabolic types: slow-twitch (type I) and fast-twitch (type II) based on the myosin heavy chain (MHC) expression and on the oxidative/glycolytic metabolic pathway utilized for ATP production [10]. Slow-twitch and fast-twitch fibers are innervated by small-caliber axons and large-caliber axons, respectively. Interestingly, the number of large-caliber axons (innervating type II fibers) is significantly lower in the spinal cord of ALS patients and of the SOD1G85R transgenic mouse model, than in controls, whereas the number of small-caliber axons (innervating type I fibers) is maintained [11,12]. Indeed, selected muscles, including the extrinsic eye muscles and the bladder detrusor muscle, are selectively spared in ALS, suggesting that the corresponding motor neurons in charge of their innervation are relatively resistant to neurodegeneration. Consistently with these data, in the ALS mouse model, muscles enriched in slow-twitch fibers undergo denervation at later stages, compared with those housing higher numbers of fast-twitch fibers [13]. These pieces of evidence suggest that, during the early stages of ALS pathogenesis, the muscle fibers and the motor neurons innervating them closely collaborate to counteract the skeletal muscle atrophy. Intrinsic properties of muscle cells, together with the pattern of impulse activity imposed on the muscle fibers, play a leading role in determining fiber type composition in the regenerating muscles, which in turn responds differently to the same stimulation pattern.

MicroRNAs are important regulators of gene expression through a post-transcriptional mechanism, and, through the binding of a microRNA to its specific target, can promote mRNA stabilization or degradation or can repress translation. A key factor in this regulatory mechanism is the RNA-induced silencing complex (RISC), a cytoplasmic ribonucleoprotein complex that incorporates the mature microRNA and uses it as a template to recognize its target transcript [14,15]. The targeting then occurs by the complementary binding of a small region of the microRNA, usually 7 nucleotides long, called seed sequence, and the 3′ untranslated region (UTR) of the mRNA. Unlike the regulation of gene expression mediated by transcriptional factors, which appears to be “on or off”, microRNAs tend to modulate the expression of target genes on a continuous trend, so they are referred to as “fine tuners” [16]. Although the effect of a single microRNA on the expression levels of a specific transcript may appear small, the combinatory effects of different microRNAs on a same target, or on several targets within the same signaling pathway, could become noteworthy [17,18]. MicroRNAs are involved in a broader range of biological processes, both physiological and pathological, and their dysregulation is involved in several human diseases. Many microRNAs are ubiquitously expressed and hence play pleiotropic roles, while others display tissue-specific expression and functions. In addition to being widely proven as molecular biomarkers in many conditions, they have also been considered as promising therapeutic targets.

Considering the involvement of skeletal muscle in ALS pathogenesis, muscle microRNAs could be regarded as relevant players modulating the course of the disease.

The aim of this review is to summarize how specific microRNAs are altered in the skeletal muscle of ALS mouse models and patients, and how this dysregulation could participate and interfere with the balance between denervation/re-innervation and muscle regeneration/atrophy processes.

## 2. MicroRNA Signaling Network in the Skeletal Muscle

The skeletal muscle is a very adaptive tissue with remarkable regenerative capacities. After muscle injury, satellite cells, mononucleated muscle stem cells, normally quiescent and located between the sarcolemma and the basement membrane of muscle fibers, start to proliferate and differentiate into myotubes to form new tissue and repair the musculature. A set of microRNAs enriched and specifically expressed in the skeletal muscle have been identified and designated as myomiRs: miR-1, miR-133a, miR-133b, miR-206, miR-208a, miR-208b, miR-499, and miR-486 [19,20,21,22,23]. MyomiRs fully take part in the molecular network regulating myogenesis and muscular regeneration processes by targeting several myogenic transcription factors and controlling the progression of myogenic differentiation (see Nie et al. [24] for a review). Also, the conversion from fast to slow fibers could be controlled by specific myomiRs, which regulate the expression of myosin types during muscle atrophy. In particular, miR-208b and miR-499 play redundant roles in the specification of muscle fiber identity by activating slow-twitch and repressing fast-twitch myofiber gene programs [22].

In ALS, the activation of satellite cells at the level of the neuromuscular junction promotes tissue regeneration and the reorganization of the muscular fibers to counteract denervation. The role of microRNA in the control of these crucial mechanisms has been investigated, though not yet conclusively clarified. Several studies on muscle biopsies and on in vitro primary satellite cell cultures have been carried out to define the role of microRNAs during the denervation process, as described below. It could hence be interesting and useful to determine if the altered expression of specific microRNAs in the skeletal muscle of patients could contribute to the disease course and represent a reactive mechanism to counteract muscular atrophy. If this is the case, it would be reasonable to design and test microRNA-targeted therapies to sustain this mechanism, with the aim of promoting muscle regeneration.

## 3. MicroRNAs Altered in the Skeletal Muscle of ALS Mouse Models

Williams and colleagues [25] originally investigated microRNA expression in the skeletal muscle during ALS progression. They showed that miR-206 is dramatically upregulated in muscle tissues of symptomatic G93A-SOD1 transgenic mice (expressing a mutant form of human *SOD1* gene, in which glycine 93 is changed to alanine) compared with controls; they also observed the downregulation of miR-133a, miR-133b, and miR-1 and the upregulation of miR-23a and miR-23b (Table 1) [25]. In this model, the upregulation of miR-206 coincided with the onset of neurological symptoms, since the transcriptional activation of miR-206 was activated in response to skeletal muscle denervation by the myogenic basic helix-loop-helix (bHLH) proteins MyoD and myogenin [25]. miR-206 mediated its effects by suppressing muscular histone deacetylase 4 (HDAC4) protein levels. HDAC4 inhibition, in turn, induced the expression of FGFBP1, which promoted re-innervation and regeneration within the neuromuscular junction [25]. Moreover, the same authors demonstrated that upon denervation miR-206 levels increased with different rates between fast and slow muscles. In particular, upon lower limb denervation in wild-type mice, miR-206 transcript levels robustly increased in muscles that contain predominantly fast-twitch fibers, namely, extensor digitorum longus (EDL), tibialis anterior (TA), and gastrocnemius/plantaris (G/P). Since the soleus (SOL) contains predominantly slow-twitch myofibers and higher levels of miR-206, in this muscle the upregulation of miR-206 was less substantial after denervation [25]. The authors concluded that miR-206 is required for an efficient regeneration of neuromuscular synapses after acute nerve injury, slowing down ALS progression in the G93A-SOD1 mouse model.

Toivonen and colleagues later defined, by microarray analyses, how the microRNA expression profile was affected in vulnerable (fast, i.e., EDL) and resistant (slow, i.e., SOL) muscle types of symptomatic G93A-SOD1 mice in comparison with wild type mice [26]. Among those microRNAs identified as dysregulated in various age/gender/muscle groups (namely, miR-206, miR-1, miR-133a, miR-133b, miR-145; see Table 1), miR-206 was the only one consistently altered in the skeletal muscle during the disease pathology [26], confirming the results of Williams et al. [25]. Moreover, they observed that miR-206 was increased in fast-twitch muscles with respect to the slow ones, increasing its expression in the most severely affected animals. Since miR-206 was also augmented in the circulation of symptomatic animals, as well as in a small group of ALS patients tested in this paper, the authors defined it as a promising candidate biomarker for ALS [26].

In line with these forms of evidence, Valdez and colleagues indicated miR-206 as the prevalent microRNA regulating the repair of the neuromuscular junction following nerve injury in G93A-SOD1 mouse model [31]. In fact, by means of selective deletion of miR-206, the authors demonstrated its unique role in stress responses at the neuromuscular junction [31].

Interestingly, both TDP-43 and FUS/TLS, mutated in familiar ALS, encode RNA-binding proteins involved in multiple steps of RNA processing and associated with components of microRNAs biogenesis pathway [32]. King and co-workers demonstrated that TDP-43 physically associates with the mature forms of the miR-1/miR-206 family of microRNAs in muscle cells, but not with the co-transcribed miR-133 [33]. TDP-43 physically prevents the interaction between miR-1 and miR-206 with the RISC complex. In the skeletal muscle, the dampening activity of these microRNAs results in elevated levels of their protein targets (i.e., insulin-like growth factor 1 (IGF-1) and HDAC4) [33]. This experimental evidence suggested an alternative mechanism of regulation in which a selective interaction between a mature microRNA and a protein limits the activity of mature microRNAs, independently from their transcription or biogenesis [33].

## 4. MicroRNAs Expression in Skeletal Muscle of Patients

So far, few studies have investigated the role of skeletal muscle microRNAs in the regulation of myogenesis, neuromuscular junction innervation, and fiber type switch processes in ALS patients.

Following the above discussed results obtained by Williams and collaborators [25], Bruneteau and colleagues investigated the involvement of the miR-206/HDAC4 pathway in the compensatory muscle reinnervation mechanism in ALS patients [27]. To assess its possible contribution to the prognostic variability, they analyzed a single time-point and classified patients into two groups based on disease progression [27]. MiR-206 transcripts were significantly upregulated in deltoid muscle specimens of ALS patients compared with control subjects, with an increased trend in long-term survivor patients (>5 years of disease progression without requiring respiratory support or gastronomy feeding), although the difference between this group and the group of patients with rapidly progressive disease did not result statistically significant [27].

Russell and co-workers demonstrated that, compared with healthy controls, in addition to miR-206, also miR-23a, miR-29b, miR-31, and miR-455 were increased in the skeletal muscle of ALS patients (Table 1); miR-1 and miR-181 levels were elevated, but not statistically significant, potentially due to the relative small sample size [28]. They focused particularly on miR-23a, which was also dysregulated in the mouse model [25], and demonstrated that it repressed PGC-1α (peroxisome proliferator-activated receptor gamma coactivator 1-alpha) translation acting on the 3′ UTR [28]. They also demonstrated that transgenic mice over-expressing miR-23a had a reduction in PGC-1α, cytochrome-b, and COXIV (cytochrome C oxidase subunit 4) protein levels [28], proteins involved in mitochondrial biogenesis and function, fusion, and electron transport chain activity. Thus, these results showed that the mitochondrial dysfunction observed in the skeletal muscle of ALS patients was associated with a reduction in PGC-1α signaling networks; also, the increase in several microRNAs could be potentially implicated in skeletal muscle and neuromuscular junction regeneration. The authors concluded that the therapeutic inhibition of miR-23a might be a strategy to rescue PGC-1α activity and ameliorate skeletal muscle mitochondrial function in ALS [28]. Interestingly, miR-23a/PGC-1α can also control the muscle fiber type determination and oxidative metabolism, being expressed preferentially in muscle enriched in type I fibers [34,35].

Later on, Jensen et colleagues [30] studied the degeneration/regeneration process in a time-course perspective by analyzing skeletal muscle biopsies from ALS patients collected before and after a 12-week period of normal daily activities and comparing with healthy age-matched control tissues. They demonstrated that miR-1, miR-26a, miR-133a, and miR-455 were reduced in ALS patients compared with controls, suggesting that both proliferation and myogenic differentiation might be altered [30] (Table 1). These microRNAs are indeed involved in the regulation of different processes during skeletal muscle development: miR-1, for instance, is required for muscle differentiation by acting on HDAC4 and PAX7, a transcription factor important for satellite cells’ function, while miR-133 promotes proliferation [24]. These data were consistent with those obtained in the mouse model by Williams et al. and Taivonen et al. regarding miR-1 and miR-133a [25,26], highlighting additional shared mechanisms between murine models and humans.

Despite the numerous data emerging from these studies, their heterogeneity somehow underlines the difficulty in translating results from animal models; in fact, ALS is a very complex disease with a pleiotropic molecular etiopathogenesis, and which depends upon several different species-specific variables. In order to assess the skeletal muscle response during ALS progression, we have analyzed in our lab the expression of selected genes and microRNAs in patients, considering the duration and evolution of the disease [36]. The gene expression pattern was first evaluated in relation with the disease duration in patients categorized into “slow” or “rapid” groups, showing more or less than 4 years of disease progression, respectively. The results indicate that miR-133a, miR-29c, miR-9, and miR-208b were significantly upregulated in the ALS slow group, whereas miR-1 and miR-208b expression was lower in the rapid group, relative to controls [36] (Table 2). Interestingly, the expression of miR-499, miR-29c, and miR-208b changed significantly between the two ALS groups [36] (Table 3). The downregulation of miR-208b and miR-499 observed in the rapid versus slow progression groups was known to be associated with fiber type switch in the skeletal muscles. We then comparatively analyzed gene expression data in patients stratified based on disease duration at biopsy (early: less than one year; late: more than one year). In this case, the expression of miR-206, miR-155, miR-23a, miR-133a, miR-133b, miR-29c, and miR-9 increased only in the early stage group, compared with controls [36] (Table 2). miR-206 and miR-9 expression levels also significantly changed between the early and the late groups (Table 3); interestingly, miR-206 levels inversely correlated with the time from symptoms onset to muscle biopsy [36]. Conversely, miR-9 expression did not correlate with the progression of the disease. These data indicated that the skeletal muscle of ALS patients activated an early response to denervation, which decreased over time at later stages.

At the same time, Pegoraro and collaborators analyzed the expression levels of muscle-specific microRNAs in patients and controls, taking into account disease duration and the age at onset of symptoms [29]. Specifically, patients were divided in subgroups according to their gender (males, females), the age at onset of symptoms (early onset: before 55 years; late onset: next 55 years), and the duration of the disease (short duration: 0–6 months; long duration: 7–36 months). miR-1, miR-206, miR-133a, miR-133b, miR27a, miR-155, miR-146a, and miR-221 were upregulated in the skeletal muscle of ALS patients compared with controls, and the expression levels of these microRNAs were higher in male patients than in female patients, except for miR-1 [29]. The levels of microRNAs analyzed were significantly higher in early onset than late onset patients, and significantly lower, except for miR-133b, in patients with short versus long disease duration [29] (Table 3).

Another recent study compared the expression of small RNAs in muscle tissues of ALS patients and healthy age-matched controls by means of small RNA-Seq [37]. The authors identified 758 un-annotated and 134 annotated tags (including microRNAs, snoRNAs, and mtRNAs) differentially expressed in the skeletal muscle of patients, and several of the dysregulated microRNAs have previously been linked with ALS [37]. Also in this study, the patients were divided into two groups on the basis of disease progression, with the aim of identifying any group-specific molecular markers that could be associated with disease severity. Among the 19 microRNAs identified in patients showing a defined molecular signature compared with controls, only miR-133a and miR-27a were in common with previous data [37] (Table 2).

Finally, in vitro cultures of satellite cells from ALS patients and mouse models were also analyzed and some characteristics of these cells have been shown, as a senescent-like morphology, disturbed differentiation, and an apparent inability to proceed through the myogenic program [36,38,39,40], resulting in a decreased ability to regenerate and mature to functional myofibers. Few works have tried to clarify if the modification of these processes was accompanied by variations in the expression levels of selected microRNAs. A significant downregulation of miR-133, miR-206, and miR-1 was demonstrated in vitro, during the differentiation time course of C2C12/SOD1G93A cells, a murine myoblast cell line stably expressing SOD1-G93A mutation, compared to control C2C12 cells [41].

## 5. Discussion

**Figure 1:**
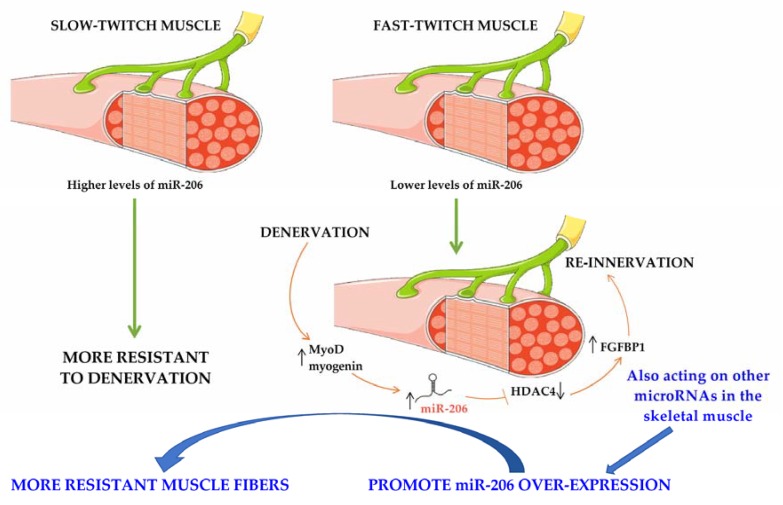
miR-206 signaling in slow and fast twitch muscles. miR-206 levels are higher in slow muscles than in fast ones and this basal expression makes the slow-twitch muscles more resistant to denervation. During the denervation process, typical of ALS disease, in fast-twitch muscles miR-206 levels increase to sustain re-innervation. Acting directly on microRNAs in the skeletal muscle could be a feasible therapeutic strategy aimed at making the muscle more resistant to denervation, increasing the responses that promote re-innervation (figure modified from https://smart.servier.com/). The green arrows link the different responses of slow- and fast-twitch muscles to the different miR-206 expression levels; the orange arrows show the signaling cascade in fast-twitch muscle following denervation; ↑: increased levels; ↓: decreased levels.

The experimental data obtained to date indicate the reproducible alteration of specific microRNAs (miR-206, miR-133a, miR-133b, miR-1, miR-23a, and miR-455) in the skeletal muscles of ALS patients and in the murine model of ALS (Table 1), yet the results concerning other microRNAs, such as miR-133a, miR-133b, miR-1, and miR-455, are sometimes contrasting and poorly reproducible. The strongest data are those concerning miR-206. Indeed, the mechanisms leading to the increase of miR-206 are apparently conserved in the skeletal muscle of the mouse model and in patients, and its upregulation is a response to the denervation specific to ALS. The importance of considering the type of muscle analyzed in each work can also be observed, as fast and slow muscles respond differently to denervation. miR-206 expression is highly enriched in slow muscles, which are in fact more resistant to denervation in the mouse models of ALS [42] (Figure 1).

As expected, different myomiRs results are altered and plausibly implicated in the molecular pathogenesis of ALS, though data concerning other “non-muscular” microRNAs also appear interesting. Additional in-depth analyses may be needed to clarify the complex network of epigenetic post-transcriptional mechanisms underlying ALS pathogenesis. This approach may highlight additional disease-specific pathways, thus enabling a better understanding of ALS pathophysiology and the discovery of novel biomarkers through a bottom-up approach.

It is certainly easier to obtain more reproducible data in the mouse models, since it is possible to work on scalable numbers of replicates and samples, overcoming the limitations in size that is inherent to the studies on patients. Muscle biopsy is unfortunately an invasive practice and the lack of homogeneity of the samples in terms of age, course of disease, site of sampling, and muscle types further complicates the analysis of data. Stratifying patients and analyzing the results considering different clinical variables (Table 2 and Table 3) could represent a successful strategy to evaluate the specific mechanisms involved in the pathogenesis of ALS. In fact, microRNAs such as miR-499 and miR-208b appear involved only when the duration of the disease is taken into account [36].

Despite the clinical and genetic heterogeneity of ALS the resulting neuromuscular phenotypes are largely overlapping. Hence, focusing primarily on patients by taking into account all clinical variables would be a desirable aim for future experimental studies. This approach may allow for an improved understanding of how the human body compensates for the disease, hence suggesting and testing novel strategies to sustain and boost these physiologic self-healing responses. In this regard, increasing the number of patients included in study samples, by supporting and fostering collaborative multicenter studies, might allow for the identification of specific molecular signatures that enable the categorization of patients based on muscular microRNA profiles, used as reliable disease biomarkers for prognostic purposes.

## 6. Conclusions

Pharmacological interventions focused on preventing or halting motor neuron degeneration in ALS have been unsuccessful and there is an urgent need for innovative therapeutic approaches. Given that the current chances to find a cure for ALS may be low, symptomatic treatments such as riluzole are the only plausible therapeutic remedies. The derangement of the functional cross-talk between neuronal and non-neuronal cells could make some therapeutic strategies ineffective. The availability of data about key regulators of the retrograde signaling in the skeletal muscle of ALS patients would represent a shortcut to the clinical context. There is a complex molecular network acting in the skeletal muscle of ALS mouse models and patients, and miR-206 emerges as an important common microRNA implicated in different processes during ALS progression. A valuable strategy to delay ALS progression could target the muscular molecular pathways in which the mentioned microRNAs are involved, supporting the re-innervation process, with the aim of improving motor performance.

## Figures and Tables

**Table 1:** Dysregulated microRNAs in amyotrophic lateral sclerosis (ALS) skeletal muscle.

MicroRNA	Model	Change	Reference
**miR-206**	G93A-SOD1 mouse	↑	[25]
	G93A-SOD1 mouse	↑	[26]
	Human	↑	[27]
	Human	↑	[28]
	Human	↑	[29]
**miR-133a**	G93A-SOD1 mouse	↓	[25]
	G93A-SOD1 mouse	↓	[26]
	Human	↓	[30]
	Human	↑	[29]
**miR-133b**	G93A-SOD1 mouse	↓	[25]
	G93A-SOD1 mouse	↓	[26]
	Human	↑	[29]
**miR-1**	G93A-SOD1 mouse	↓	[25]
	G93A-SOD1 mouse	↓	[26]
	Human	↓	[30]
	Human	↑	[29]
**miR-23a**	G93A-SOD1 mouse	↑	[25]
	Human	↑	[28]
**miR-23b**	G93A-SOD1 mouse	↑	[25]
**miR-145**	G93A-SOD1 mouse	↓	[26]
**miR-29b**	Human	↑	[28]
**miR-31**	Human	↑	[28]
**miR-455**	Human	↑	[28]
	Human	↓	[30]
**miR-26a**	Human	↓	[30]
**miR-27a**	Human	↑	[29]
**miR-155**	Human	↑	[29]
**miR-146a**	Human	↑	[29]
**miR-221**	Human	↑	[29]

↑ up-regulated microRNA in ALS compared with control samples; ↓ down-regulated microRNA in ALS compared with control samples.

**Table 2:** Dysregulated microRNAs in specific groups of ALS patients with respect to controls.

MicroRNA	Patients’ Group	Reference
**miR-133a**	↑ in patients with a slow disease progression ^1^	[36]
**miR-29c**		
**miR-9**		
**miR-208b**		
**miR-1**	↓ in patients with a rapid disease progression ^2^	[36]
**miR-208b**		
**miR-206**	↑ in “early” skeletal muscle samples ^3^	[36]
**miR-155**		
**miR-23a**		
**miR-133a**		
**miR-133b**		
**miR-29c**		
**miR-9**		
**miR-100**	Dysregulated in a group of ALS patients ^4^	[37]
**miR-1291**		
**miR-1303**		
**miR-133a**		
**miR-150**		
**miR-199**		
**miR-27a**		
**miR-3607**		
**miR-378**		
**miR-378d**		
**miR-424**		
**miR-450a**		
**miR-450b**		
**miR-486**		
**miR-501**		
**miR-502**		
**miR-542**		
**miR-660**		
**miR-855**		

↑ up-regulated; ^1^ ≥4 years of duration of disease without requiring respiratory supports; ↓ down-regulated; ^2^ <4 years of disease progression without respiratory support or death occurring <4 years from symptoms onset; ^3^ less than one year from symptoms onset to muscle biopsy; ^4^ patients with a higher disease severity.

**Table 3:** Dysregulated microRNAs in stratified ALS patients.

MicroRNA	Patients’ Group	Reference
**miR-499**	↑ in patients with a slow disease progression ^1^ compared with rapid patients ^2^	[36]
**miR-29c**		
**miR-208b**		
**miR-1**	↑ in patients with a higher disease duration ^3^ compared with rapid patients ^4^	[29]
**miR-206**		
**miR-133a**		
**miR-27a**		
**miR-155**		
**miR-146**		
**miR-221**		
**miR-1**	↑ in early onset patients ^5^ compared with late onset patients ^6^	[29]
**miR-206**		
**miR-133a**		
**miR-27a**		
**miR-155**		
**miR-146**		
**miR-221**		
**miR-206**	Decreased during muscular atrophy process ^7^	[36]
**miR-9**		

↑ up-regulated; ^1^ ≥4 years of duration of disease without requiring respiratory supports; ^2^ <4 years of disease progression without respiratory support or death occurring <4 years from symptoms onset; ^3^ 7–36 months of disease duration; ^4^ 0–6 months of disease duration; ^5^ <55 years at onset; ^6^ >55 years at onset; ^7^ Based on Pearson’s correlation test of microRNAs relative expression levels of ALS patients with the time from symptoms onset to muscle biopsy.

## Abbreviations

ALS	Amyotrophic Lateral Sclerosis
SOD1	Superoxide Dismutase 1
TARDBP	TAR DNA-Binding Protein
UBQLN2	Ubiquilin 2
FUS	Fused in Sarcoma
ALSFRS-R	ALS Functional Rating Scale Revised
MHC	Myosin Heavy Chain
ATP	Adenosine Triphosphate
mRNA	Messenger RNA
RISC	RNA-Induced Silencing Complex
UTR	Untranslated Region
myomiRs	Muscle Specific MicroRNAs
bHLH	Basic Helix-Loop-Helix
MyoD	Myogenic Differentiation
HDAC4	Histone Deacetylase 4
FGFBP1	Fibroblast Growth Factor Binding Protein 1
EDL	Extensor Digitorum Longus
TA	Tibialis Anterior
G/P	Gastrocnemius/Plantaris
SOL	Soleus
TDP-43	TAR DNA-Binding Protein 43
FUS/TLS	FUS RNA Binding Protein
IGF-1	Insulin-like Growth Factor 1
PGC-1α	Peroxisome Proliferator-Activated Receptor Gamma Coactivator 1-Alpha
3′UTR	3′ Untranslated Region
COXIV	Cytochrome C Oxidase Subunit 4
PAX7	Paired Box 7
RNA-Seq	RNA Sequencing
snoRNAs	Small Nucleolar RNAs
mtRNAs	Mitochondrial RNAs
